# Predictive model of the occurrence of sepsis-associated encephalopathy in sepsis patients based on the combination of IFN-γ, TNF-α and CD4^+^/CD8^+^ ratio

**DOI:** 10.1097/MD.0000000000045287

**Published:** 2025-10-17

**Authors:** Enqi Tian, Yan Guo, Yufeng Chen, Wenjuan Li, Xiaofeng Zhang, Guoping Wang

**Affiliations:** a Department of Anaesthesia, Changzhi People’s Hospital, Changzhi City, Shaanxi Province, China.

**Keywords:** CD4+/CD8+ ratio, IFN-γ, predictive model, sepsis, sepsis-associated encephalopathy, TNF-α

## Abstract

Sepsis-associated encephalopathy (SAE) is a significant clinical challenge in sepsis patients, contributing to prolonged hospitalization and increased mortality. The dysregulated immune response and neuroinflammatory processes have been implicated in its pathogenesis. This study aimed to develop a predictive model for SAE based on a combination of immunological markers. A retrospective study was conducted at a single center from January 2020 to December 2023, involving 98 sepsis patients. Clinical, laboratory, and immunological parameters were analyzed. Statistical analyses included chi-square tests, Wilcoxon rank-sum tests, logistic regression to identify independent risk factors, and receiver operating characteristic curve analysis to evaluate the predictive performance of the combined model. The encephalopathy group had a significantly higher prevalence of pulmonary disease, vasopressor use, and elevated body temperature. Key laboratory findings included significantly lower levels of interferon-gamma (IFN-γ) (*P* < .001), tumor necrosis factor-alpha (TNF-α) (*P* < .001), and a decreased CD4+/CD8 + ratio (*P* = .001) compared to the non-encephalopathy group. The logistic regression model confirmed these immunological markers as independent risk factors. The combined model of IFN-γ, TNF-α, and the CD4+/CD8 + ratio demonstrated a high predictive value with an AUC of 0.845. The combination of IFN-γ, TNF-α, and CD4+/CD8 + ratio provides a validated model for predicting SAE. These findings suggest that incorporating these immunological markers could improve risk stratification and early intervention. Future prospective, multicenter studies are recommended to validate this model for routine clinical practice.

## 1. Introduction

Sepsis is a life-threatening condition characterized by a dysregulated host response to infection, leading to multiple organ dysfunction and significant morbidity and mortality rates worldwide.^[1,2]^ Globally, sepsis affects an estimated 49 million people each year, resulting in approximately 11 million deaths, thereby representing a major public health burden. The incidence of sepsis continues to rise, particularly in aging populations and low-to-middle-income countries.^[3]^ Among the myriad complications associated with sepsis, sepsis-associated encephalopathy (SAE) represents a complex neurological manifestation that contributes to prolonged hospitalization, increased healthcare costs, and heightened mortality risk.^[4]^ It is estimated that SAE occurs in 30 to 70% of sepsis patients, with higher prevalence rates observed in those with severe sepsis and septic shock. The presence of SAE is associated with a mortality rate of up to 40 to 60%, and survivors often experience long-term cognitive impairment and functional disability, underscoring the critical need for early prediction and intervention.^[5–7]^

A key characteristic of SAE is its potential reversibility if identified and managed early during the course of sepsis.^[8,9]^ However, its onset can be insidious, often developing within the first 24 to 48 hours after the diagnosis of sepsis, making timely recognition a challenge in clinical practice.^[10]^ This early window of opportunity, coupled with the grave outcomes associated with SAE, provides a strong rationale for developing robust predictive tools that can identify high-risk patients at the earliest stage, thereby enabling preemptive interventions to mitigate neurological damage and improve overall prognosis.^[11]^ While the pathophysiology of SAE remains incompletely understood, accumulating evidence suggests that immune dysregulation and neuroinflammatory processes play pivotal roles in its development and progression.^[12,13]^ The intricate interplay of systemic inflammation, immune dysfunction, and neuroinflammatory cascades underscores the multifaceted nature of SAE, necessitating a comprehensive approach to its recognition, understanding, and management.^[14]^ Dysregulation of proinflammatory cytokines, such as interferon-gamma (IFN-γ) and tumor necrosis factor-alpha (TNF-α), plays a critical role in the pathophysiology of SAE. IFN-γ, as a pivotal proinflammatory cytokine, modulates immune responses and has been implicated in the pathogenesis of sepsis.^[15]^ It has been suggested that dysregulated production of IFN-γ contributes to immune suppression and subsequent susceptibility to secondary infections and organ dysfunction in sepsis.^[16]^ TNF-α, another essential cytokine in sepsis, exerts pleiotropic effects on the immune system and has been linked to the pathogenesis of tissue injury and multi-organ dysfunction in sepsis. The aberrant production of TNF-α has been associated with blood-brain barrier disruption and neuroinflammation, which are central to the development of SAE.^[17,18]^

Dysregulated cytokine profiles and altered T cell homeostasis have been implicated in the pathogenesis of SAE, contributing to neuroinflammation, blood-brain barrier disruption, and neuronal dysfunction.^[19,20]^ Moreover, clinical and laboratory markers, including disease severity scores, biochemical parameters, and hematological indices, have shown promise as predictive indicators for the occurrence of SAE.^[21,22]^ Integrating immunological, clinical, and laboratory parameters into a comprehensive predictive model holds potential for enhancing risk stratification, early identification, and targeted interventions for SAE. In light of the high incidence and severe consequences of SAE, there is an urgent clinical need for reliable predictive tools that can identify high-risk patients at an early stage. Despite the exploration of numerous predictive models for SAE, the incorporation of clinical scores, routine laboratory parameters, and novel biomarkers remains predominant.^[23–25]^ While these models contribute to risk stratification, there remains a need for models that specifically capture the profound immune dysregulation characteristic of sepsis and SAE. For instance, recent models have focused on infectious pathogens or combinations of inflammatory markers,^[26]^ but few have integrated the specific interplay of key cytokines (IFN-γ, TNF-α) and T-cell homeostasis (CD4^+^/CD8^+^ ratio) into a unified predictive tool. In this context, the present study aims to establish a predictive model for the occurrence of SAE in sepsis patients based on the combination of IFN-γ, TNF-α and the CD4^+^/CD8^+^ ratio.

## 2. Materials and methods

### 2.1. Study design and population

This single-center, retrospective cohort study was conducted and reported in accordance with the STROBE statement.^[27]^ The study included a total of 98 sepsis patients admitted to our hospital from 1^st^ January 2020 to 31^st^ December 2023. The diagnosis of sepsis was established according to the Chinese Expert Consensus on Early Prevention and Intervention of Sepsis.^[28]^ This consensus defines sepsis as a life-threatening organ dysfunction caused by a dysregulated host response to infection. It aligns with the international Sepsis-3 criteria by requiring a suspected or confirmed infection and an acute increase of ≥2 points in the sequential organ failure assessment (SOFA) score.^[29]^ The patients were divided into 2 groups based on the presence or absence of septic encephalopathy: the occurrence group (n = 26) and the nonoccurrence group (n = 72). The diagnosis of SAE was characterized by an alteration in consciousness, which was assessed daily during sepsis using the intensive care unit (ICU) confusion assessment method for the ICU in patients with sepsis.^[30]^ Moreover, SAE was diagnosed only when the encephalopathy was not attributable to any other primary etiology (e.g., direct intracranial infection, stroke, metabolic derangements not related to sepsis, sedative overdose, or preexisting dementia). All data were extracted from the hospital’s electronic medical record system, which serves as the comprehensive clinical data warehouse for all inpatient and laboratory records. This data source was deemed suitable as it captures all required variables for this study.

Inclusion criteria were as follows: Aged 18 years or older; Initial diagnosis of sepsis and treatment received at our hospital within the study period; and Full compliance with treatment and examinations. Exclusion criteria were designed to rule out alternative causes of encephalopathy and confounding factors: preexisting underlying brain diseases (e.g., known dementia, Parkinson disease, history of stroke with residual deficit) before the onset of sepsis; Coexisting psychiatric disorders such as depression or schizophrenia; Presence of malignant tumors, severe underlying cardiac, hepatic, or renal dysfunction, or other conditions that may independently affect mental status or prognosis; Pregnancy or lactation; Presence of hematologic or rheumatic immune system diseases; Use of steroids or immunosuppressants prior to admission; and Incomplete clinical data.

This study was approved by the Ethics Committee of Changzhi People’s Hospital. The procedures were conducted in accordance with the ethical standards set forth by the Committee on Human Experimentation and the Helsinki Declaration of 1964, as revised in 2013. Informed consent was waived by the Ethics Committee of Changzhi People’s Hospital for this retrospective study due to the exclusive use of de-identified patient data, which posed no potential harm or impact on patient care. This waiver was approved by the institutional review board and ethics committee of our institution in accordance with regulatory and ethical guidelines pertaining to retrospective studies.

### 2.2. Sample size calculation

The final cohort comprised 98 patients, including the occurrence group (n = 26) and the nonoccurrence group (n = 72). A post hoc power analysis using G*Power (version 3.1) indicated 82% power (α = 0.05) to detect a 25% absolute difference in primary efficacy outcomes (CD4^+^/CD8^+^) between groups, accounting for the observed sample sizes and an anticipated 15% attrition rate.

### 2.3. Data collection

This includes age, gender, body mass index, smoking history, alcohol consumption history, as well as comorbidities such as hypertension, diabetes, hyperlipidemia, other neurological disorders, hypothyroidism, liver disease, coagulation disorders, Acute Physiology and Chronic Health Evaluation II (APACHE II) scores, simplified acute physiology score II scores, onset to neuro-symptom confirmation time (ONCT) and so on, which were collected and recorded from the medical record system. Laboratory data included routine blood measurements, liver and renal function, arterial blood gas analysis, procalcitonin (PCT), blood urea nitrogen, C-reactive protein (CRP), neuron-specific enolase, S100 calcium binding protein β (S100β) and interleukin (IL)-6. Peripheral blood was drawn to measure S100 Calcium Binding Protein A8, TNF receptor associated factor 6, IL-1β, IFN-γ and TNF-α. Finally, hospital and ICU stay, bacteriological categories, and 28-day mortality were also recorded in all the enrolled patients.

### 2.4. Blood examination

Blood examination was performed within 24 hours following the diagnosis of sepsis. A fasting 5mL blood sample was collected from the antecubital vein in the morning for blood testing. The sample was centrifuged at a speed of 3000 r/min for 5 minutes, and the supernatant was used for the analysis of aspartate aminotransferase (AST), serum creatinine (Scr), PCT, TNF-α, IFN-γ, monocyte chemoattractant protein-1 and IL-10. Scr was analyzed using the Roche cobas c702 fully automatic biochemical analyzer. PCT, TNF-α, IFN-γ, monocyte chemoattractant protein-1 and IL-10 were tested via enzyme-linked immunosorbent assay using reagent kits obtained from Shanghai Enzyme-linked Biological Technology Co., Ltd. C-reactive protein was measured using a nephelometry assay with a Siemens BNII or BN Pro specific protein analyzer (Siemens, Germany) and matched reagents (batch number 16573C). Measurements of D-dimer, prothrombin time (PT), fibrinogen, and international standardized ratio were conducted using the Stago-4 fully automatic coagulation analyzer (manufacturer: Diagnostica Stago, France). Platelet count was determined using the Beckman DxH800 hematology analyzer.

### 2.5. Statistical analysis

There were no missing data for the primary outcome or the key predictor variables (IFN-γ, TNF-α, CD4^+^/CD8^+^ ratio) as these were essential for the study. For other variables with missing data, a complete-case analysis approach was employed, and these cases were excluded from specific analyses where data was missing. For categorical data, [n (%)] was used for representation. The chi-square test was applied with the basic formula when the sample size was ≥40 and the theoretical frequency *T* ≥ 5, with the test statistic represented by χ^2^. When the sample size was ≥40 but the theoretical frequency 1 ≤ *T* < 5, the chi-square test was adjusted using the correction formula. In cases where the sample size was <40 or the theoretical frequency *T* < 1, statistical analysis was conducted using Fisher exact probability method. For normally distributed continuous data, the formatwas employed as mean ± standard deviation. Non-normally distributed data was analyzed using Wilcoxon rank-sum test. *P* < .05 were considered as statistical significance. Univariable logistic regression was first performed. The indicators that showed significant differences in the univariable analysis were included as covariates in a multivariable logistic regression analysis to identify independent risk factors for SAE. The diagnostic performance of the combined model of IFN-γ, TNF-α, and the CD4^+^/CD8^+^ ratio in distinguishing SAE was evaluated using the area under the receiver operating characteristic (ROC) curve. All statistical analyses were performed using SPSS 25.0 statistical software (SPSS Inc., Chicago).

## 3. Results

### 3.1. Sociodemographic characteristics

Based on the sociodemographic characteristics of the patient cohort, there were no significant differences in age, gender distribution and admission type including emergency, elective and urgent (*P* > .05) (Table 1). In the encephalopathy group, patients had a mean age of 52 years and were predominantly male. In the no encephalopathy group, patients had a mean age of 55 years.

**Table 1:** Sociodemographic characteristics of sepsis patients with and without sepsis-associated encephalopathy.

Characteristic	Encephalopathy group (n = 26)	No encephalopathy group (n = 72)	*t*/χ^2^	*P*
Age (yr)	52.11 ± 18.60	54.60 ± 12.80	0.630	.533
Gender				
Male	18 (69.23%)	40 (55.56%)	0.967	.325
Female	8 (30.76%)	32 (44.44%)		
Admission type				
Emergency	8 (30.77%)	16 (22.22%)	0.363	.547
Elective	16 (61.54%)	50 (69.44%)	0.243	.622
Urgent	2 (7.69%)	6 (8.33%)	0.000	1.000

### 3.2. Clinical data

The encephalopathy group had a significantly longer median duration of neurological symptoms and signs before enrollment, (median onset to neuro-symptom confirmation time: 2.70 ± 0.03 days vs 2.68 ± 0.04 days, *P* = .036). The prevalence of pulmonary disease was markedly higher in the encephalopathy group (65.38% vs 15.28%, *P* < .001). A significantly higher proportion of patients in the encephalopathy group presented with a body temperature of ≥39°C (92.31% vs 68.06%, *P* = .030) (Table 2). Notably, although not statistically significant, the encephalopathy group showed lower mean systolic blood pressure (systolic blood pressure: 159.31 ± 39.30 mm Hg vs 168.60 ± 36.90 mm Hg, *P* = .300) and diastolic blood pressure (diastolic blood pressure: 93.60 ± 15.80 mm Hg vs 99.76 ± 20.91 mm Hg, *P* = .125) compared to the non-encephalopathy group. These trends may partly explain the higher requirement for vasopressor support in the encephalopathy group, as hypotension is a common indicator for vasopressor use in septic patients. Regarding disease severity scores, the encephalopathy group presented with slightly but significantly higher SOFA scores (5.27 ± 0.32 vs 5.12 ± 0.28, *P* = .038), APACHE II scores (89.76 ± 2.97 vs 88.39 ± 2.06, *P* = .038), and simplified acute physiology score II scores (43.16 ± 1.54 vs 42.39 ± 1.79, *P* = .042). Physiologically, the encephalopathy group also demonstrated a higher heart rate (95.50 ± 9.89 vs 90.55 ± 11.75 beats/min, *P* = .043) and respiratory rate (24.58 ± 5.56 vs 21.50 ± 4.84 breaths/min, *P* = .017), reflecting a more pronounced systemic inflammatory response and potential compensatory mechanisms. Other parameters, including body mass index, smoking history, drinking history, part of comorbidities, recent surgery, recent transfusion, mechanical ventilation, blood pressure, and initial glasgow coma scale scores, did not show statistically significant differences between the 2 groups (*P* > .05).

**Table 2:** Comparison of clinical data of the patients between the 2 groups.

Characteristic	Encephalopathy group (n = 26)	No encephalopathy group (n = 72)	*t*/χ^2^	*P*
BMI (kg/m^2^)	23.65 ± 0.89	23.79 ± 0.76	0.717	.478
Smoking history	12 (46.15%)	35 (48.61%)	0.000	1.000
Drinking history	10 (38.46%)	21 (29.17%)	0.394	.530
Median ONCT (d)	2.70 ± 0.03	2.68 ± 0.04	2.145	.036
Comorbidities				
Cardiovascular diseases	6 (23.08%)	31 (43.06%)	2.450	.118
Peripheral vascular disease	1 (3.85%)	2 (2.78%)	0.000	1.000
Other neurological diseases	1 (3.85%)	3 (4.17%)	0.000	1.000
Hypertension	6 (23.08%)	16 (22.22%)	0.000	1.000
Pulmonary disease	17 (65.38%)	11 (15.28%)	21.109	<.001
Diabetes	3 (11.54%)	5 (6.94%)	0.100	.752
Hypothyroidism	2 (7.69%)	4 (5.56%)	0.000	1.000
Liver disease	1 (3.85%)	1 (1.39%)	0.000	1.000
Coagulopathy	2 (7.69%)	2 (2.78%)	0.257	.612
Clinical disease severity score				
SOFA score	5.27 ± 0.32	5.12 ± 0.28	2.150	.038
APACHE II score	89.76 ± 2.97	88.39 ± 2.06	2.163	.038
SAPS II score	43.16 ± 1.54	42.39 ± 1.79	2.089	.042
Recent management				
Recent surgery	11 (42.31%)	23 (31.94%)	0.506	.477
Recent transfusion	7 (26.92%)	27 (37.50%)	0.534	.465
Mechanical ventilation	12 (46.15%)	26 (36.11%)	0.444	.505
Vasopressor	15 (57.69%)	16 (22.22%)	9.533	.002
Vital signs				
Body temperature ≥ 39°C	24 (92.31%)	49 (68.06%)/	4.705	.030
Heart rate (beats/min)	95.50 ± 9.89	90.55 ± 11.75	2.077	.043
Respiratory rate (breaths/min)	24.58 ± 5.56	21.50 ± 4.84	2.49	.017
SBP (mm Hg)	159.31 ± 39.30	168.60 ± 36.90	1.049	.300
DBP (mm Hg)	93.60 ± 15.80	99.76 ± 20.91	1.556	.125
Initial GCS scores	10.19 ± 1.40	10.12 ± 1.40	0.199	.843

The patients with SIRS without infection.

APACHE II = acute physiology and chronic health evaluation II, BMI = body mass index, DBP = diastolic blood pressure, GCS = glasgow coma scale, ONCT = onset to neuro-symptom confirmation time, SAPS II = acute physiology score II, SBP = systolic blood pressure, SOFA = sequential organ failure assessment.

### 3.3. Laboratory tests

**Figure 1.**
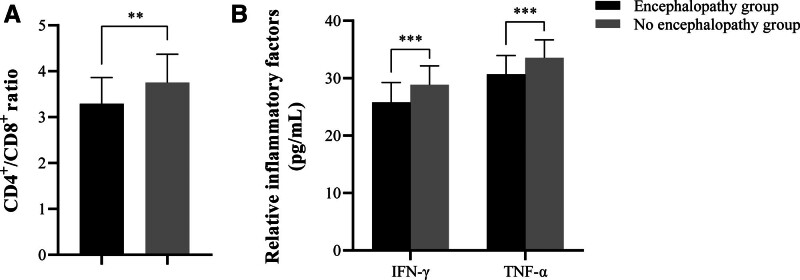
Comparison of IFN-γ, TNF-α and CD4^+^/ CD8^+^ ratio between encephalopathy group and no encephalopathy group. (A) The levels of IFN-γ and TNF-α between 2 groups. ***P < .001. (B) The CD4^+^/CD8^+^ ratio between 2 groups. **P < .01. IFN-γ = interferon-gamma, TNF-α = tumor necrosis factor-alpha.

The encephalopathy group demonstrated significantly higher lactate levels (1.52 ± 0.03 mmol/L vs 1.50 ± 0.04 mmol/L, *P* = .008) and lower arterial pH (7.32 ± 0.04 vs 7.35 ± 0.04, *P* = .007), indicating poorer tissue perfusion and metabolic acidosis. Unexpectedly, the encephalopathy group had a higher partial pressure of oxygen (PO_2_: 109.59 ± 13.67 mm Hg vs 103.17 ± 12.69 mm Hg, *P* = .043), this might reflect differences in supplemental oxygen therapy or early respiratory support strategies in more critically ill patients. Hepatic and renal function markers were notably altered, with the encephalopathy group showing higher alanine aminotransferase (alanine aminotransferase: 178.67 ± 6.79 U/L vs 182.15 ± 8.53 U/L, *P* = .042), creatinine (1.21 ± 0.04 K/µL vs 1.23 ± 0.04 K/µL, *P *= .007), and bilirubin (0.54 ± 0.03 EU/dL vs 0.56 ± 0.04 EU/dL, *P* = .001). Hematological assessment revealed a lower platelet count (226.98 ± 9.79 K/µL vs 231.97 ± 8.67 K/µL, *P* = .027) and a reduced mean corpuscular volume (MCV: 94.72 ± 2.78 fL vs 96.04 ± 2.17 fL, *P* = .035) in the encephalopathy group. Furthermore, the level of the brain injury biomarker S100β was significantly higher in the encephalopathy group (2.03 ± 0.18 μg/L vs. 2.15 ± 0.29 μg/L, *P* = .020) (Table 3). Most importantly, and central to the aim of this study, the levels of key immunological markers were significantly dysregulated in the encephalopathy group (Fig. 1). This group displayed markedly lower levels of IFN-γ (*P* < .001), TNF-α (*P* < .001), and a decreased CD4^+^/CD8^+^ ratio (*P* = .001) compared to the no encephalopathy group. In contrast, no statistically significant differences were found between the groups for the following parameters: PCO_2_, PCT, CRP, Scr, neuron-specific enolase, blood urea nitrogen, AST, hemoglobin, potassium, sodium, PT, red cell distribution, white blood cell, lymphocyte percentage, or neutrophil percentage (*P* > .05).

**Table 3:** Comparison of laboratory tests between the 2 groups.

Characteristic	Encephalopathy group (n = 26)	No encephalopathy group (n = 72)	*t*	*P*
Arterial blood gases				
Lactate (mmol/L)	1.52 ± 0.03	1.50 ± 0.04	2.731	.008
PCO_2_ (mm Hg)	40.01 ± 2.99	40.02 ± 3.19	0.019	.985
PO_2_ (mm Hg)	109.59 ± 13.67	103.17 ± 12.69	2.090	.043
pH	7.32 ± 0.04	7.35 ± 0.04	2.853	.007
Inflammation markers				
PCT (mg/L)	70.12 ± 5.45	71.87 ± 6.20	1.335	.188
CRP (mg/L)	113.52 ± 18.08	123.87 ± 17.65	1.335	.192
Liver function tests				
AST (U/L)	1.50 ± 0.24	1.51 ± 0.29	0.199	.843
Bilirubin (EU/dL)	0.54 ± 0.03	0.56 ± 0.04	3.417	.001
ALT (U/L)	178.67 ± 6.79	182.15 ± 8.53	2.086	.042
Kidney function tests				
Serum creatinine (mmlol/L)	216.70 ± 25.76	225.78 ± 22.90	1.585	.121
Creatinine (K/µL)	1.21 ± 0.04	1.23 ± 0.04	2.826	.007
BUN (K/µL)	23.01 ± 2.95	23.00 ± 2.17	0.018	.986
Brain injury biomarkers				
S100β (μg/L)	2.03 ± 0.18	2.15 ± 0.29	2.389	.020
NSE (ng/mL)	41.57 ± 0.87	41.72 ± 0.81	0.799	.429
Hematological indices				
Hemoglobin (g/dL)	11.50 ± 2.27	11.49 ± 2.19	0.011	.991
Platelet (K/µL)	226.98 ± 9.79	231.97 ± 8.67	2.296	.027
PT (s)	14.09 ± 0.49	13.86 ± 0.37	2.166	.037
RDW (%)	14.81 ± 2.17	14.70 ± 2.64	0.211	.834
WBC (K/µL)	10.60 ± 2.41	10.80 ± 2.33	0.374	.710
Lymphocyte (%)	9.09 ± 1.47	9.54 ± 1.33	1.364	.180
Neutrophil (%)	81.28 ± 4.87	81.15 ± 4.44	0.122	.904
MCV (fL)	94.72 ± 2.78	96.04 ± 2.17	2.191	.035
Electrolytes				
Potassium (K/µL)	4.10 ± 0.85	4.19 ± 0.94	0.482	.632
Sodium (K/µL)	138.02 ± 15.98	138.01 ± 15.47	0.001	.999

ALT = alanine aminotransferase, AST = aspartate aminotransferase, BUN = blood urea nitrogen, CRP = C-reactive protein, MCV = mean corpuscular volume, NSE = neuron-specific enolase, PCT = procalcitonin, PT = prothrombin time, RDW = red cell distribution width, S100β = S100 calcium binding protein β, WBC = white blood cell.

### 3.4. Factors Predictive of SAE

A higher SOFA score (OR = 0.158, *P* = .029) and a higher APACHE II score (OR = 0.782, *P* = .016) were predictive of SAE. Several vital signs and clinical interventions were significant predictors, including the presence of pulmonary disease (OR = 0.095, *P* < .001), the use of vasopressors (OR = 0.210, *P* = .001), a body temperature ≥ 39°C (OR = 0.178, *P* = .026), and an elevated respiratory rate (OR = 0.883, *P* = .013). Key laboratory abnormalities were also strongly predictive. These included higher lactate levels (OR = 0.000, *P* = .022), lower arterial pH (OR = 29,008,735.045, *P* = .006), higher creatinine (OR = 4,243,289.424, *P* = .012), higher bilirubin (OR = 29,059,859.818, *P* = .008), lower platelet counts (OR = 1.062, *P* = .021), longer PT (OR = 0.259, *P* = .020), and lower mean corpuscular volume (MCV) (OR = 1.261, *P* = .020). Crucially, lower levels of IFN-γ (OR = 1.314, *P* < .001) and TNF-α (OR = 1.320, *P* < .001) measured within 24 hours of sepsis diagnosis were associated with the occurrence of SAE during the ICU stay. Similarly, a lower CD4^+^/CD8^+^ ratio (OR = 3.747, *P* = .003) was also an independent predictive factor (Table 4).

**Table 4:** Logistic regression predictive model of sepsis-associated encephalopathy in sepsis patients.

Characteristic	OR	95% CI	*P*
Median ONCT	0.000	(0.000–0.001)	.075
Disease severity scores			
SOFA score	0.158	(0.031–0.802)	.029
APACHE II score	0.782	(0.641–0.954)	.016
Comorbidities			
Pulmonary disease	0.095	(0.028–0.322)	<.001
Recent management			
Vasopressor	0.210	(0.079–0.559)	.001
Vital signs			
Body temperature ≥ 39°C	0.178	(0.038–0.832)	.026
Respiratory rate	0.883	(0.797–0.978)	.013
Arterial blood gases			
Lactate	0.000	(0.000–0.001)	.022
PO_2_	0.962	(0.927–0.998)	.037
pH	29,008,735.045	(1,241,853.147–701274568.546)	.006
Kidney function tests			
Creatinine (K/µL)	4,243,289.424	(156,154.477–120569852.661)	.012
Liver function tests			
Bilirubin (EU/dL)	29,059,859.818	(1,854,056.425–478431673.108)	.008
Hematological indices			
Platelet (K/µL)	1.062	(1.009–1.118)	.021
PT (s)	0.259	(0.081–0.831)	.020
MCV (fL)	1.261	(1.036–1.535)	.020
Immunological markers			
IFN-γ (pg/mL)	1.314	(1.142–1.512)	<.001
TNF-α (pg/mL)	1.320	(1.138–1.531)	<.001
CD4^+^/CD8^+^	3.747	(1.582–8.874)	.003

APACHE II = acute physiology and chronic health evaluation II, CI = confidence interval, IFN-γ = interferon-gamma, MCV = mean corpuscular volume, ONCT = onset to neuro-symptom confirmation time, OR = odds ratio, PT = prothrombin time, SOFA = sequential organ failure assessment, TNF-α = tumor necrosis factor-alpha.

### 3.5. Combined model

**Figure 2.**
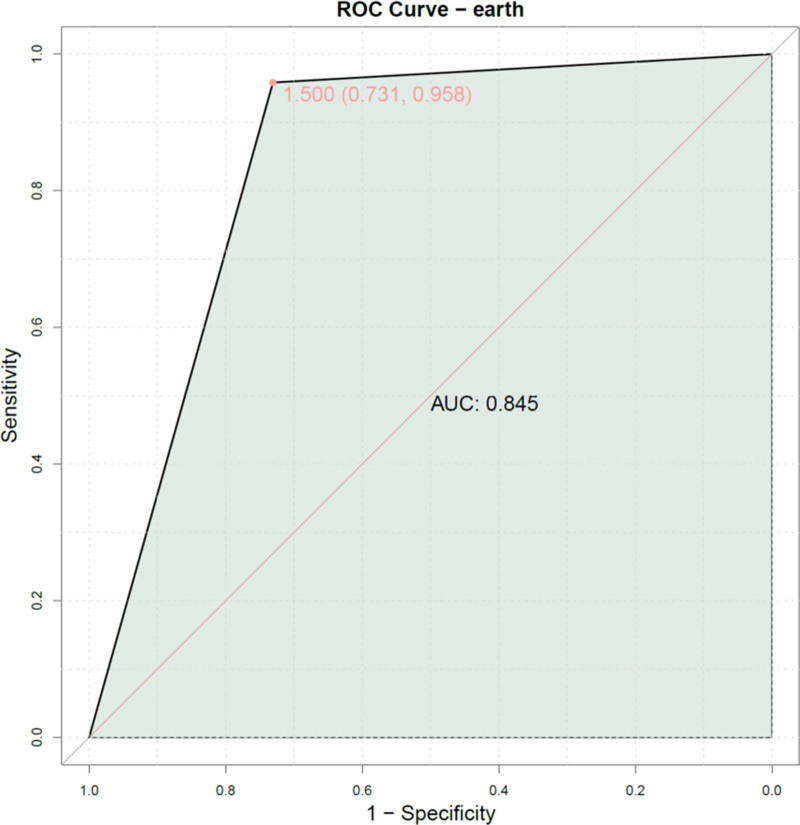
The combined model of IFN-γ, TNF-α, and CD4^+^/CD8^+^ ratio. IFN-γ = interferon-gamma, TNF-α = tumor necrosis factor-alpha.

The predictive values of IFN-γ, TNF-α, and the CD4^+^/CD8^+^ ratio were combined to establish a joint model. This combination was based on their strong statistical significance in univariate and multivariate analysis. ROC analysis demonstrated that the combined model yielded an area under the curve (AUC) value of 0.845 (Fig. 2), indicating significant predictive value for the occurrence of SAE.

## 4. Discussion

This study aimed to develop and validate a predictive model for SAE based on a combination of immunological markers. First, patients who developed SAE presented with distinct clinical features, including a higher prevalence of pulmonary disease, greater vasopressor usage, and elevated body temperature. Second, the encephalopathy group exhibited more severe laboratory derangements, including higher lactate levels, metabolic acidosis, and signs of hepatic/renal impairment. Third, and most critically, we identified significant dysregulation in key immunological markers, with the SAE group demonstrating markedly lower levels of IFN-γ, TNF-α, and a decreased CD4^+^/CD8^+^ ratio. Finally, a logistic regression model confirmed these immunological markers as independent risk factors, and their combination yielded a predictive model with high discriminatory power.

Our analysis revealed that specific clinical markers, such as preexisting pulmonary disease, vasopressor requirement, and pyrexia (≥39°C), were strongly associated with the development of SAE. This aligns with the understanding that the severity of the initial septic insult and the degree of cardiovascular compromise are pivotal in the pathogenesis of end-organ dysfunction, including encephalopathy.^[4,5]^ The higher requirement for vasopressors indicates more profound circulatory failure, which can compromise cerebral perfusion and contribute to ischemic and inflammatory brain injury. The association with pulmonary disease may reflect a higher burden of inflammatory cytokines from a pulmonary source or hypoxia, both of which can exacerbate neuroinflammation. Our findings corroborate previous studies that have linked illness severity scores and hemodynamic instability to worse neurological outcomes in sepsis,^[4,21]^ but we specifically highlight the predictive value of these readily available clinical assessments at the bedside.

The SAE group demonstrated significant metabolic disturbances, including higher lactate levels and lower arterial pH, indicative of tissue hypoperfusion and a more severe septic state. Furthermore, we observed greater impairment in hepatic and renal function markers. These findings are consistent with the nature of SAE as a manifestation of multi-organ dysfunction rather than an isolated neurological event.^[31,32]^ The elevated S100β level, a biomarker of astrocyte activation and blood-brain barrier disruption, further supports the presence of underlying brain injury in these patients. While previous research has established a correlation between overall organ failure and SAE,^[31]^ our study quantifies the specific laboratory abnormalities that accompany SAE, providing a clearer information of the associated systemic metabolic and organ dysfunctions that clinicians can monitor. Although not all clinical parameters reached statistical significance, the encephalopathy group consistently trended towards a worse clinical condition. For instance, we observed lower mean systolic and diastolic blood pressures and a significantly higher requirement for vasopressor support. This overall trend suggests that the development of SAE is multifactorial, driven by a confluence of hemodynamic instability, inflammatory, and metabolic insults.^[4,6,33,34]^ Our model, by focusing on the most statistically robust predictors, captures key elements of this pathophysiology. However, clinicians should consider the aggregate burden of illness, including these non-significant trends, when assessing individual patient risk for SAE.

The most salient finding of our study was the significant downregulation of IFN-γ and TNF-α, alongside a reduced CD4^+^/CD8^+^ ratio, in patients with SAE. This appears counterintuitive given the pro-inflammatory nature of these cytokines but is a hallmark of the immunosuppressive phase of sepsis, which is increasingly recognized as a critical driver of secondary infections and organ dysfunction.^[16,35,36]^ Lower levels of IFN-γ and TNF-α may signify a state of immune exhaustion, where the innate and adaptive immune systems are unable to mount an effective response. This immune paralysis can facilitate the unchecked spread of inflammation and microbial products, ultimately damaging the brain. Similarly, a decreased CD4^+^/CD8^+^ ratio reflects T-cell dysfunction and imbalance, which has been directly linked to poor outcomes and encephalopathy in sepsis.^[33,37]^ While others have noted immune dysregulation in sepsis broadly, our study novelly identifies this specific triad of immunological markers (IFN-γ, TNF-α, CD4^+^/CD8^+^ ratio) as a key and independent predictor of SAE, moving beyond mere association to propose a actionable predictive tool.

The combination of IFN-γ, TNF-α, and the CD4^+^/CD8^+^ ratio into a single model achieved an AUC of 0.845, demonstrating superior predictive performance. This multi-marker approach is strengths-based as it captures different but complementary aspects of the septic immune response: cytokine release and T-cell homeostasis. This aligns with the current paradigm that SAE is a multifactorial disorder and that a single biomarker is insufficient for accurate prediction.^[19,36]^ Our model’s performance suggests that assessing the immune phenotype early in sepsis could significantly enhance risk stratification for SAE. This approach is novel compared to previous models that have primarily relied on clinical scores or nonspecific inflammatory markers like CRP and PCT,^[21,22]^ offering a more specific insight into the pathological immune mechanisms driving brain dysfunction.

The findings of this study carry significant clinical implications for the early identification and management of SAE. The validated predictive model based on a combination of clinical, laboratory, and immunological parameters can aid clinicians in risk stratification, prompt recognition of encephalopathy, and targeted interventions to mitigate its impact on patient outcomes. By identifying patients at high risk for SAE, healthcare providers can potentially optimize management strategies, including neurological monitoring, tailored immunomodulatory therapies, and supportive care, with the goal of reducing the burden of encephalopathy and improving overall patient prognosis. Despite the valuable insights gained from this study, some limitations should be acknowledged. The retrospective nature of the study and the utilization of de-identified patient data may have introduced inherent biases and limitations related to data availability and completeness. It is noteworthy that our cohort was predominantly male (59.2%) with a high proportion of elective admissions (67.3%). While this demographic and admission pattern did not show a statistically significant difference between groups, it may influence the generalizability of our findings. Epidemiological studies suggest that sex-based differences in immune response may influence sepsis outcomes, though findings regarding SAE specifically are still emerging.^[38,39]^ The high rate of elective admissions (e.g., for post-operative care) might indicate a study population with different baseline risks and etiologies of sepsis compared to those studies enrolling predominantly from emergency departments.^[40,41]^ This highlights the need for external validation of our model in broader, more diverse sepsis populations. Additionally, the study was conducted at a single center, which may limit the generalizability of the findings to broader patient populations and diverse healthcare settings. Furthermore, the study’s sample size and the specific patient cohort may have influenced the observed associations and the predictive model’s performance. Future research endeavors should aim to address these limitations by conducting prospective multicenter studies with larger and more diverse patient cohorts to validate the predictive model’s performance and generalizability. Moreover, while the study provides valuable insights into the predictive modeling of SAE, additional research was warranted to further elucidate the underlying pathophysiological mechanisms and the intricate interplay between immune dysregulation and neurological manifestations in sepsis. Comprehensive investigations into the molecular and cellular pathways involved in SAE, incorporating advanced immunological and neuroimaging techniques, may yield deeper insights and potential targets for novel therapeutic interventions. Furthermore, prospective studies were needed to validate the clinical utility of the predictive model in real-world clinical practice, assess its impact on patient outcomes, and refine its applicability in different healthcare settings.

## 5. Conclusion

In summary, this study developed and validated a predictive model for SAE that integrates the immunological markers IFN-γ, TNF-α, and the CD4^+^/CD8^+^ ratio. The combined immunological model demonstrated superior predictive performance, which is competitive. The implications of this study are 2-fold. For clinical practice, our model proposes a promising tool for early risk stratification of SAE using biomarkers that reflect its core pathophysiology. For future research, the findings must be validated in a large-scale, prospective, multicenter cohort study to confirm their robustness and generalizability. Such a study should incorporate serial measurements of these immunological parameters to better understand their trajectory and refine the predictive model. If validated, this model could serve as a basis for future interventional trials targeting immune dysfunction to prevent or mitigate SAE.

## Acknowledgments

The authors express their appreciation to staff in Changzhi People’s Hospital, for their technical assistance.

## Author contributions

**Conceptualization:** Enqi Tian, Guoping Wang.

**Data curation:** Enqi Tian, Yan Guo, Yufeng Chen, Wenjuan Li, Xiaofeng Zhang, Guoping Wang.

**Formal analysis:** Enqi Tian, Yan Guo, Yufeng Chen, Wenjuan Li, Xiaofeng Zhang, Guoping Wang.

**Investigation:** Yan Guo, Yufeng Chen, Wenjuan Li, Xiaofeng Zhang.

**Methodology:** Yan Guo, Yufeng Chen, Wenjuan Li, Xiaofeng Zhang.

**Project administration:** Guoping Wang.

**Supervision:** Guoping Wang.

**Writing – original draft:** Enqi Tian, Guoping Wang.

**Writing – review & editing:** Enqi Tian, Guoping Wang.
